# Between the Sponge and the Tap—Bacterial Communities at Overlooked Hospital Hygiene Hotspots

**DOI:** 10.3390/microorganisms14030552

**Published:** 2026-02-28

**Authors:** Marek Ussowicz, Monika Rosa, Kornelia Gajek, Anita Brzoza, Tomasz Jarmoliński, Anna Panasiuk, Elżbieta Wawrzyniak-Dzierżek, Łukasz Łaczmański

**Affiliations:** 1 Department of Pediatric Bone Marrow Transplantation, Oncology and Hematology, Wroclaw Medical University, 50-367 Wrocław, Poland; 2 Hirszfeld Institute of Immunology and Experimental Therapy, Polish Academy of Sciences, 53-114 Wrocław, Poland; 3 Department of Pediatric Bone Marrow Transplantation, Oncology and Hematology, University Clinical Hospital, 50-556 Wrocław, Poland

**Keywords:** hospital microbiome, sponges, faucets, 16S rRNA amplicon sequencing, infection control

## Abstract

Hospital environments host diverse microbial communities that may contribute to nosocomial infections. Moisture-retaining surfaces such as cleaning sponges and faucet edges represent high-contact, under-investigated hygiene hotspots, particularly in wards caring for immunocompromised patients. Environmental samples were collected from cleaning sponges (n = 14) and faucet edges (n = 4) across multiple hospital rooms of a paediatric haematology–oncology unit, with domestic physician sponges as controls (n = 3). DNA was extracted and sequenced targeting the V3–V4 and V7–V9 hypervariable regions of the 16S rRNA gene on the Illumina MiSeq platform. Taxonomic composition and alpha/beta diversity were assessed using QIIME 2 and R. Sponge samples were dominated by Moraxellaceae, particularly *Acinetobacter* and *Enhydrobacter*, and showed significantly lower alpha diversity than faucet samples (Shannon index: Kruskal–Wallis H = 8.4, *p* = 0.01; Faith’s phylogenetic diversity: H = 9.17, *p* = 0.01). Faucet samples were enriched in human-associated genera including *Staphylococcus*, *Streptococcus*, and *Chryseobacterium*. Statistically significant beta-diversity differences were detected between sponge and faucet communities by PERMANOVA based on Bray–Curtis dissimilarity (*p* = 0.01), whereas no significant clustering by room or floor location was observed (*p* = 0.29). Potentially pathogenic taxa including *Aeromonas*, *Pseudomonas*, and Enterobacteriaceae were identified across both surface types. Domestic control sponges showed distinct microbiome profiles from hospital samples. Microbial communities differ significantly between hospital sponges and faucets, with surface type rather than location as the primary determinant of community structure. The presence of opportunistic pathogens on both surface types highlights the importance of enhanced hygiene protocols, inclusion of faucet edges and sink drains in routine decontamination schedules, and regular microbiological surveillance in clinical settings caring for immunocompromised patients.

## 1. Introduction

Hospital environments represent complex ecosystems where microbial communities persist on a variety of surfaces, often despite routine cleaning and disinfection protocols [1]. While the environmental presence of microorganisms is inevitable, the composition and diversity of microbial populations are of critical importance, particularly in clinical settings where immunocompromised individuals are at heightened risk for opportunistic infections [2,3].

Faucets and cleaning sponges are among the most frequently touched and moisture-retaining surfaces in healthcare facilities. Their materials and usage patterns create favourable niches for microbial colonisation, including potentially pathogenic and antimicrobial-resistant species [4]. Recent evidence has established that sink drains and faucet-adjacent surfaces harbor not only diverse microbial communities but also a considerable reservoir of antimicrobial resistance genes (ARGs) and multidrug-resistant organisms. Hospital sink drains have been characterized as a melting pot for horizontal antimicrobial resistance (AMR) gene transfer between environmental and nosocomial pathogens, and ICU sinks have been shown to be persistently colonized with MDR bacteria carrying mobilizable resistance plasmids [5,6]. Anantharajah et al. documented a long-term ICU outbreak of carbapenemase-producing organisms associated specifically with contaminated sink drains [7]. Kelly et al. performed large-scale characterisation of hospital wastewater microbiomes and clinical isolates, identifying high degrees of resistome overlap between plumbing biofilms and patient isolates—providing a key mechanistic link between environmental surfaces and patient infections [8]. Hanafiah et al. reviewed insights into microbiome and ARGs from hospital environmental surfaces, supporting the concept that high-touch, moisture-prone surfaces are prime AMR amplification zones [9].

Sponges, in particular, are supposed to accumulate biofilms and support the growth of resilient microorganisms, making them a possible vector for microbial transmission if not replaced regularly or adequately sterilised [10,11,12,]. The microbiome of kitchen sponges has been characterised extensively in domestic settings: Moraxellaceae (particularly *Acinetobacter*, *Enhydrobacter*, and *Moraxella*), Pseudomonadaceae, and *Chryseobacterium* consistently dominate sponge communities across Europe, while viable pathogens such as *Acinetobacter baumannii* and *Klebsiella pneumoniae* have been confirmed in sponge microbiomes [10,12,,13].

Likewise, faucet handles and edges often harbour skin- and water-associated bacteria, especially in high-contact zones [14,15]. Faucet handles and edges in clinical settings have been shown to harbour human-associated skin bacteria and waterborne taxa, but a direct intra-ward comparison with sponge microbiomes across multiple hypervariable 16S rRNA regions has not previously been reported from a paediatric haematology–oncology unit [16,17].

Culture-independent methods such as 16S rRNA gene sequencing have revolutionised the study of environmental bacterial communities, allowing for a comprehensive and unbiased view of bacterial community structure and diversity. The use of multiple hypervariable regions (e.g., V3V4 and V7V9) further improves taxonomic resolution and enhances our understanding of microbial distribution on different surface types.

In our study, we conducted a 16S rRNA gene amplicon profiling survey of bacterial communities present on sponges and faucet surfaces from multiple hospital rooms. As a comparative control, we included sponges used by physicians in domestic environments. Our objectives were to (1) characterise the taxonomic composition of bacterial communities inhabiting these surfaces, (2) assess differences in microbial diversity between sample types and locations, and (3) identify the presence of clinically relevant taxa that may pose a risk in hospital settings.

## 2. Results

### 2.1. Initial Data Processing

#### 2.1.1. V3V4 Region of the 16S rRNA Gene

The estimated amplicon length for the V3V4 region was 444 nucleotides, with an overlap of 70 nucleotides between the paired-end reads. Forward reads were used without trimming, while reverse reads were trimmed at position 205. A total of 1307 amplicon sequence variants (ASVs) were identified. The shortest ASV was 248 nucleotides long and the longest was 446 nucleotides; 2% of the sequences were shorter than 400 nucleotides. All sequences were subsequently trimmed to 400 nucleotides in length.

#### 2.1.2. V7V9 Region of the 16S rRNA Gene

The estimated amplicon length for the V7V9 region was 392 nucleotides, with a 102-nucleotide overlap between reads. Only forward reads were used for analysis due to the low quality of reverse reads. A total of 953 ASVs were identified. The shortest variant was 249 nucleotides long and the longest was 273 nucleotides; 2% of the sequences were shorter than 250 nucleotides. Reads were subsequently trimmed to a uniform length of 249 nucleotides.

### 2.2. Taxonomic Analysis

**Figure 1:**
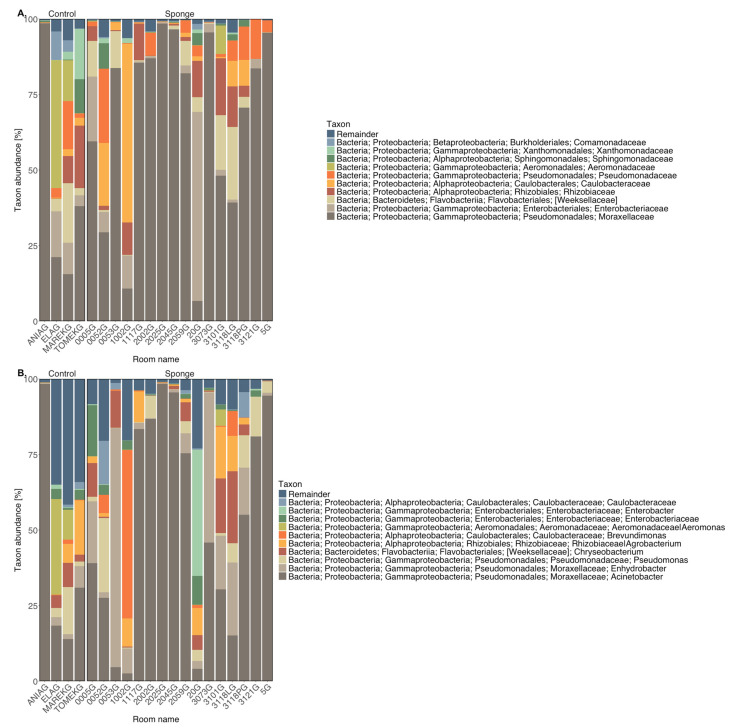
Taxonomic composition of sponge samples—most abundant taxa at the level of (**A**) families, (**B**) genera.

**Figure 2:**
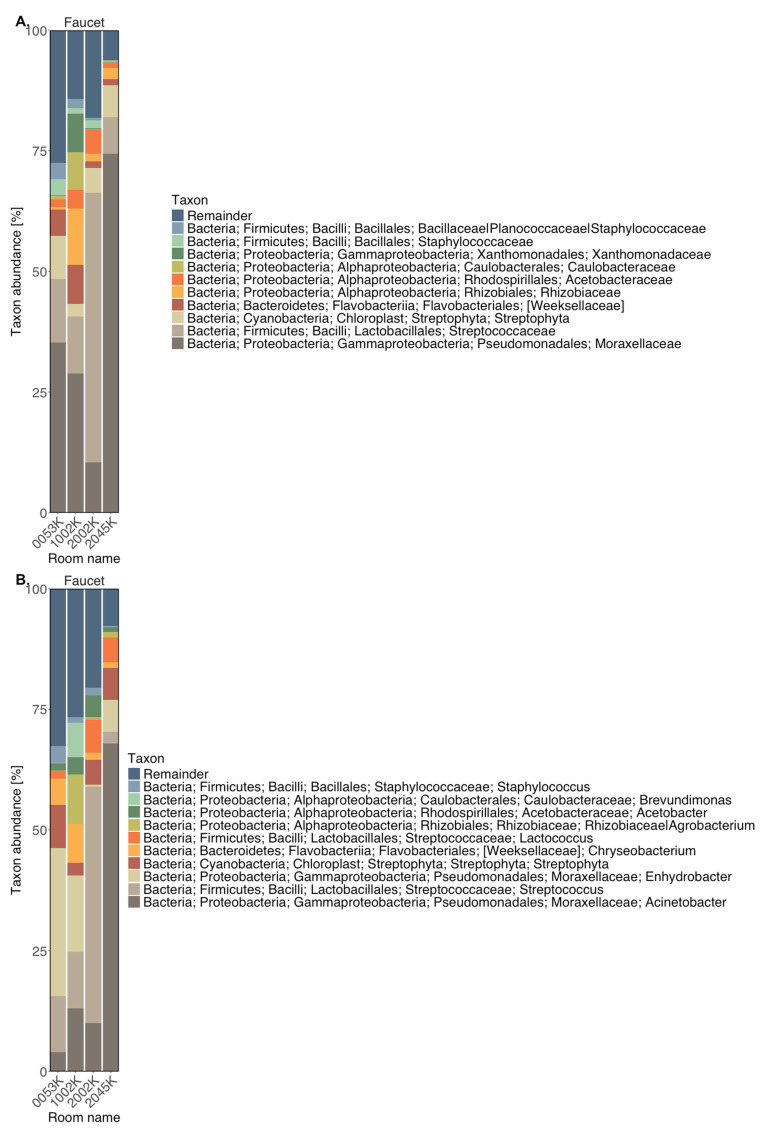
Taxonomic composition of faucet samples—most abundant taxa at the level of (**A**) families, (**B**) genera.

The 10 most abundant bacterial families and genera detected in sponge and faucet samples are shown in Figure 1 and Figure 2, respectively. Relative abundances of taxa detected in sponge and faucet samples are provided in Supplementary Tables S1 and S2.

In almost all sponge samples (except for 1002G, 20G, ELAG, and MAREKG), a substantial presence of the family Moraxellaceae was observed, particularly *Acinetobacter* and *Enhydrobacter* genera. Notably, *Enhydrobacter* was dominant in samples 0053G and 3073G. Sample 1002G had the highest abundance of *Brevundimonas* from the Caulobacteraceae family. *Aeromonas* (from Aeromonadaceae) was found in control samples ELAG and MAREKG. Interestingly, the dominant taxa in hospital sponge samples were not well reflected in control samples, except for control sample (ANIAG) with a high abundance of *Acinetobacter*, which distinguished it from the other controls. Faucet samples showed lower abundance of Moraxellaceae, although it still dominated in most. An exception was sample 2002K, in which *Streptococcus* was the dominant genus. All faucet samples contained *Staphylococcus*, especially abundant in 0053K. This sample also showed lower representation of Rhizobiaceae compared to others. Compared to sponge samples, faucet samples had a higher prevalence of *Streptococcus*, *Staphylococcus*, and *Chryseobacterium*—common skin bacterial communities.

### 2.3. Diversity Analysis

**Figure 3:**
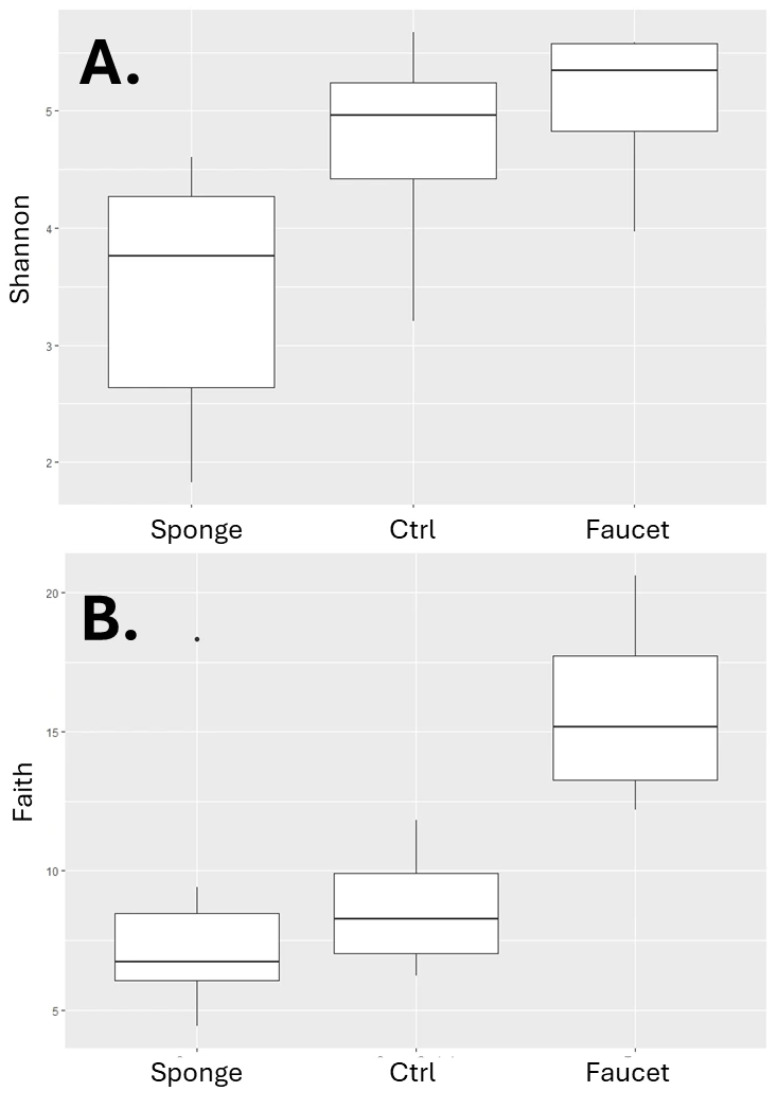
Values of (**A**) Shannon Index and (**B**) Faith’s Phylogenetic Diversity in samples grouped according to sample origin.

Faucet samples exhibited the highest within-sample microbial diversity, as measured by both Shannon index and Faith’s phylogenetic diversity (PD). Sponge samples had the lowest diversity values. Comparison of diversity indices by sample source is shown in Figure 3.

The Kruskal–Wallis test confirmed statistically significant differences in Shannon index (H = 8.4, *p* = 0.01) and Faith’s PD (H = 9.17, *p* = 0.01) between sample source groups.

Among sponge samples, MAREKG had the highest Shannon index, while 2002G had the highest phylogenetic diversity. No statistically significant differences were found between diversity indices when grouped by hospital room (Shannon index: H = 15.85, *p* = 0.46; Faith’s PD: H = 16.39, *p* = 0.42). Interestingly, most control samples had higher Shannon index values (except for ANIAG) compared to other sponge samples, although this trend was not seen for Faith’s PD.

Among faucet samples, those from floor 2 had the lowest Shannon indices, while those from floor 1 had the highest. Again, Kruskal–Wallis tests showed no significant differences by floor (Shannon index: H = 2.69, *p* = 0.26; Faith’s PD: not significant). Highest phylogenetic diversity was observed in floor 0 faucet samples; the lowest was observed in floors 1 and 2.

PERMANOVA based on Bray–Curtis dissimilarity showed no statistically significant differences between samples grouped by room (H = 1.14, *p* = 0.29), but it did detect significant differences when grouped by sample source (H = 1.45, *p* = 0.03).

**Figure 4:**
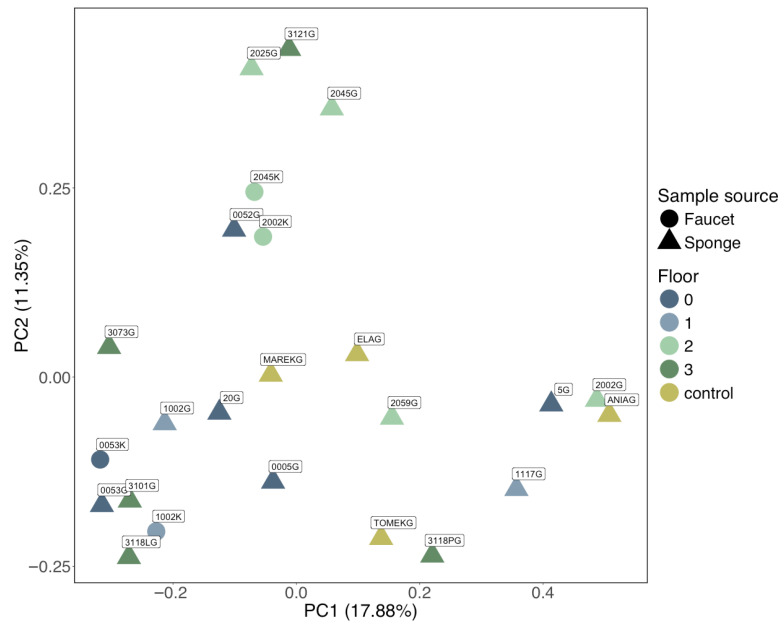
PCoA plot based on Bray–Curtis dissimilarity values.

Principal Coordinate Analysis (PCoA) of Bray–Curtis dissimilarities showed partial separation by surface type (sponges vs. faucets), whereas room/floor location did not yield a consistent pattern (Figure 4). Samples can be visually grouped into one of five groups: Group I (2002G, 3118PG), Group II (2025G, 5G, 1117G), Group III (TOMEKG, 3118LG), Group IV (3121G, 1002K, ANIAG), and Group V (other samples). However, PERMANOVA based on Bray–Curtis dissimilarity did not confirm any significant differences between these groups (H = 1.1379, *p* = 0.206).

## 3. Discussion

The built environment of hospitals represents a microbial ecosystem shaped by human activity, material composition, humidity, and cleaning practices. A recent study quantifying the burden of *Pseudomonas aeruginosa* and *Stenotrophomonas* in hospital sinks before and after environmental hygiene interventions provided empirical data supporting the clinical relevance of microbiome surveillance [18].

Our findings are broadly consistent with prior hospital surface microbiome studies that used culture-independent sequencing approaches.

Sponges demonstrated a consistent predominance of Moraxellaceae, particularly *Acinetobacter* and *Enhydrobacter* genera [12]. These bacteria are known for their ability to survive in moist environments and their role as opportunistic pathogens in immunocompromised individuals [19,20]. The presence of *Brevundimonas* in some sponge samples also warrants attention, as it has been implicated in nosocomial infections [21]. The composition of bacterial community of hospital sponges was similar to that reported in non-hospital environments (dominated by *Acinetobacter*, *Chryseobacterium*, *Enhydrobacter*, Enterobacteriaceae and *Pseudomonas*) [22]. According to Moen et al. *Acinetobacter*, *Pseudomonas*, *Enhydrobacter*, Enterobacteriaceae, *Psychrobacter*, *Chryseobacterium*, *Bacillus*, and *Staphylococcus* constitute core bacterial communities in kitchen environment [12,23]. Rampelotto et al. reported massive dominance of *Acinetobacter* and *Pseudomonas* across 663 hospital surface samples, with a homogeneous community structure characterised by low network connectedness [24].

Non-fermenting Gram-negative bacteria such as *Pseudomonas*, *Acinetobacter*, and *Chryseobacterium*, detected in the tested samples, represent a heterogeneous group of antibiotic-resistant opportunistic pathogens that are increasingly implicated in healthcare-associated infections [25]. In particular, *Acinetobacter* and *Pseudomonas* infections represent a substantial hazard for immunocompromised patients [26,27]. The dominance of *Acinetobacter* on sponge surfaces parallels findings in kitchen microbiome studies, where this genus frequently colonizes porous, wet substrates such as dishwashing sponges [28,29]. *Enhydrobacter*, although less frequently reported in clinical infections, is a common constituent of skin and environmental bacterial communities and has demonstrated resistance to desiccation and antimicrobial agents [30,31]. Control (home) sponges showed markedly different taxa than hospital sponges, indicating environment effect. Faucet samples, although less enriched in Moraxellaceae, still showed microbial profiles dominated by skin- and water-associated bacteria such as *Staphylococcus*, *Streptococcus*, and *Chryseobacterium*. Faucets and biofilms in sinks in healthcare facilities often harbor human-derived bacteria, including *Staphylococcus*, alongside waterborne taxa []. The consistent presence of these genera, which are typical members of human skin bacterial communities, suggests potential contamination from direct handling or water splashing during hand hygiene procedures.

Analysis of alpha diversity revealed significantly greater microbial richness and phylogenetic diversity in faucet samples compared to sponges. This may be attributed to the frequent contact of faucet surfaces with water from multiple sources, including human hands and cleaning activities, leading to continuous inoculation of diverse bacterial communities. In contrast, sponges, especially in hospital settings, may harbor dense but less diverse communities due to selective pressures such as desiccation resistance or antimicrobial exposure. Beta diversity analysis confirmed significant compositional differences between sponge and faucet bacterial communities, while no clear clustering was observed based on room or floor location. This indicates that surface type (sponge vs. faucet) is a stronger determinant of microbial structure than the room environment itself. Sponges and faucet handles differ markedly in material properties and exposure dynamics. Sponges are porous, retain moisture and organic residues, and undergo repeated wetting/drying cycles, creating a selective environment that can enrich fast-growing, moisture-tolerant taxa. The dominance of Moraxellaceae on sponge surfaces in our study parallels findings from kitchen and domestic environments and suggests that this family may represent a conserved coloniser of porous, wet materials regardless of environment. Conversely, the enrichment of skin-associated taxa (*Staphylococcus*, *Streptococcus*, *Chryseobacterium*) on faucet surfaces aligns with observations from ICU sink rims and contact surfaces, where frequent human hand inoculation determines community composition [17]. Faucet handles are repeatedly inoculated by hands and water splashes, and may dry, supporting a more diverse bacterial communities including skin-associated genera. The surface-type dependence, independent of room or floor location, is corroborated by the Brazilian multi-hospital ICU study which found consistent clustering by sampling site rather than geographic location [32].

The lack of clustering by room or floor implies that standard environmental parameters (e.g., air exchange, room type) are less important than direct physical use and moisture exposure. The detection of distinct sample clusters in the PCoA suggests that some surfaces harbor uniquely structured communities, potentially due to variation in cleaning frequency, sponge usage patterns, or water exposure.

Notably, several potentially pathogenic taxa were identified across both sample types, including *Staphylococcus*, *Streptococcus*, *Aeromonas*, *Pseudomonas*, and members of Enterobacteriaceae [33,34]. Non-fermenting Gram-negative bacteria such as *Pseudomonas* and *Acinetobacter* are important causes of severe infections and are frequently associated with antimicrobial resistance [35]. Several detected genera include opportunistic pathogens relevant to immunocompromised patients. *Brevundimonas* spp., although uncommon, have been reported in healthcare-associated infections (including bacteremia and device-related infections) [36,37,38,39].

These organisms are of clinical concern, as these genera are well-documented constituents of hospital plumbing biofilms and are associated with outbreaks of hospital-acquired infections, particularly in settings where patients may be vulnerable to opportunistic infections [3]. Accordingly, moist fomites and plumbing-adjacent surfaces should be considered in infection-prevention strategies for high-risk units [40,41,42]. Even with proper disinfection, up to 9.2% of recolonizing bacteria may originate from drains and 15.7% from tap water within just one week, while the continued use of sponges may undermine the effectiveness of these sanitation efforts [15]. When sponges are used in conjunction with these contaminated reservoirs, they may further compromise environmental hygiene by serving as intermediate carriers of opportunistic pathogens. Sanitization methods are not particularly effective in the case of cleaning sponges. Jacksch et al. performed metagenomic analysis of microwave-treated and untreated kitchen sponges, showing that Moraxellaceae and Pseudomonadaceae increase after sanitisation, reinforcing the view that standard decontamination may not eliminate key opportunistic genera [10]. Carstens et al. used propidium monoazide treatment to characterise viable bacterial microbiomes in kitchen sponges, confirming the presence of *Acinetobacter baumannii* and *Klebsiella pneumoniae* as dominant viable species, further emphasising pathogen viability concerns [13]. Based on these findings, we recommend replacing reusable sponges with single-use wipes or microfiber systems, including faucet handles/edges/aerators and sink drains in targeted cleaning protocols and periodic deep-cleaning, and considering targeted microbiological surveillance of moist reservoirs in high-risk areas.

Finally, hand hygiene limits touch-associated bacterial spread because hands connect patients, staff, and high-touch wet surfaces. Even when hospital surfaces harbor diverse bacteria, adherence to alcohol-based hand rub or soap-and-water interrupts transfer between fomites, patients, and the environment [43]. This is especially important for moist hotspots around sinks in high-risk units, where repeated hand contact can sustain human-associated bacterial communities.

We have to acknowledge some limitations of our study. Only four faucet-handle samples were available, which limits statistical power and the ability to generalize patterns across the ward. Faucet profiles should therefore be interpreted as exploratory and may be influenced by episodic contamination or changes in use. We also acknowledge that the use of the Greengenes 13_8 reference database may limit taxonomic resolution, particularly for rare or newly described species. However, these constraints do not undermine our main conclusions, as our interpretations focuses on differences in the overall structure and composition of bacterial communities between sample types and environments, which are robust at the genus and family levels.

## 4. Materials and Methods

### 4.1. Samples

Environmental samples were collected from the edges of faucets and from sponges located in various hospital rooms. The complete list of analysed samples is presented in Table 1. Additionally, four dishwashing sponges used by physicians in domestic settings were included as control samples.

### 4.2. Sampling Protocol

Wet cleaning sponges were sampled aseptically; the sponge was aseptically squeezed by compressing it against the inner sterile tube wall to express the retained liquid. The expressed sponge liquid (sponge eluate) was collected at the bottom of the tube and used as the analytical specimen. Faucet-handle samples were collected by swabbing with a sterile swab pre-moistened in sterile saline. Each sample was placed into a sterile tube and frozen; samples were stored at −80 °C until DNA extraction.

### 4.3. DNA Extraction

Sponge eluate samples were transferred into lysis buffer and vortexed to elute bacteria. Mechanical disruption (bead-beating) was applied to improve lysis of Gram-positive organisms. DNA was extracted using the QIAseq 16S/ITS Panels workflow (Qiagen, Hilden, Germany) following the manufacturer’s protocol. DNA concentration and purity were assessed (fluorometry and A260/280).

### 4.4. Library Preparation and PCR

Amplicon libraries were prepared using the QIAseq 16S/ITS Panels kit with primers targeting V3–V4 and V7–V9 regions. Primer sequences used for amplification of the V3V4 and V7V9 regions are listed in Table 2. PCR cycling conditions and cycle numbers were performed according to the kit protocol; negative PCR controls were included. Library size and concentration were verified prior to sequencing with TapeStation (Agilent Technologies, Santa Clara, CA, USA). Sequencing was performed on an Illumina MiSeq (Illumina, Inc., San Diego, CA, USA) platform.

### 4.5. Bioinformatic Analysis

The 16S rRNA gene amplicon sequencing analysis was performed on DNA extracted from the collected samples, amplified the V3V4 and V7V9 regions of the 16S rRNA gene. Sequencing was carried out generating raw data in the Cassava 1.8 format. The data were processed using QIIME 2 (q2cli, Amplicon and Shotgun distributions, version 2023.9) [44]. After importing the data with q2-tools, sequences were separated by 16S rRNA region and primers were removed using q2-cutadapt [45]. Sequence quality control and amplicon length estimation were performed using q2-demux. The estimated overlap between paired-end reads was also calculated. Sequence trimming, denoising, and dereplication were performed using q2-dada2 [46]. For the V3V4 region, both forward and reverse reads were used, while for the V7V9 region, only forward reads were included due to the poor quality of reverse reads. To integrate data from both sequenced regions, the SMURF algorithm implemented in q2-sidle was used [47,48]. A reference database (GreenGenes v13_8) was prepared, and the 16S rRNA gene was reconstructed from both reference and experimental sequences. Taxonomic classification was performed using the GreenGenes 13_8 (GG) reference database, as required by the q2-sidle pipeline employed for simultaneous analysis of the V3–V4 and V7–V9 hypervariable regions. Although more recent databases are available (GreenGenes2 2024.09, SILVA 138.1), compatibility with q2-sidle is limited to GreenGenes 13_8 and SILVA 128; adopting a newer database would necessitate an entirely different analytical pipeline and preclude the joint multi-region approach used here. GreenGenes was selected over SILVA 128 for the following reasons: it is one of the most widely used 16S rRNA reference databases, facilitating direct comparison with existing publications and meta-analyses; comparative evaluations have shown that GreenGenes and SILVA provide comparable reference tree quality and similar annotation error rates and GreenGenes is focused exclusively on bacteria and archaea, which aligns with the biological scope of this study and avoids classification noise introduced by the broad eukaryotic sequence content present in SILVA.

A table of amplicon sequence variants and a phylogenetic tree were generated. Taxonomic classification, alpha diversity (Shannon index and Faith’s phylogenetic diversity) [49,50], and beta diversity (Bray–Curtis dissimilarity) [51] were assessed using q2-diversity.

### 4.6. Statistical Analysis

Alpha-diversity comparisons were performed using Kruskal–Wallis tests with exact *p*-values reported, alongside non-parametric effect sizes (epsilon-squared). Beta-diversity differences were tested using PERMANOVA (Bray–Curtis; 999 permutations), reporting pseudo-F, and *p*-values. Analyses were conducted using q2-diversity; plots and PERMANOVA for PCoA were produced in R (version 4.3.2) using qiime2R (v3.4.4), ggplot2 (0.99.6 library), and vegan (v2.6-10). A *p*-value of <0.05 was considered statistically significant. Statistically significant results were visualised using box-and-whisker plots for the diversity indices: Shannon and Faith’s Phylogenetic Diversity (PD).

### 4.7. Ethics

This study involved environmental surface sampling only and did not include identifiable patient data; according to our institutional policy, formal IRB approval was not required for environmental sampling.

## 5. Conclusions

In summary, this study highlights the need for routine microbiological surveillance of overlooked hospital surfaces. The findings emphasise the role of surface material and usage patterns in shaping microbiomes within healthcare settings. It supports replacing items such as sponges with alternatives, such as single-use disposable towels or dishwashers. Routine microbiological monitoring and reconsideration of cleaning practices, especially concerning sponge use, are recommended to minimise the risk of pathogen transmission.

## Figures and Tables

**Table 1:** List of samples analysed in the experiment.

Sample ID	Sample Source	Floor/Control
0005G	Sponge	0
5G	Sponge	0
20G	Sponge	0
0052G	Sponge	0
0053G	Sponge	0
0053K	Faucet	0
1002G	Sponge	1
1002K	Faucet	1
1117G	Sponge	1
2002G	Sponge	2
2002K	Faucet	2
2025G	Sponge	2
2045G	Sponge	2
2045K	Faucet	2
2059G	Sponge	2
3073G	Sponge	3
3101G	Sponge	3
3118LG	Sponge	3
3118PG	Sponge	3
3121G	Sponge	3
ANIAG	Sponge	Control
ELAG	Sponge	Control
MAREKG	Sponge	Control
TOMEKG	Sponge	Control

**Table 2:** Primer sequences used in the study.

16S rRNA Region	Primer Type	Primer Sequence	Estimated 5′ Position on *E. coli* Genome
V3V4	Forward	CCTACGGGNGGCWGCAG	341
V3V4	Reverse	GACTACHVGGGTATCTAATCC	806
V7V9	Forward	YAACGAGCGMRACCC	1100
V7V9	Reverse	TACGGYTACCTTGTTAYGACTT	1492

## Data Availability

The original contributions presented in this study are included in the article. Further inquiries can be directed to the corresponding author.

## Supplementary Materials

The following supporting information can be downloaded at https://www.mdpi.com/article/10.3390/microorganisms14030552/s1, Table S1: Percentage abundance of Species detected in sponge samples; Table S2: Percentage abundance of Species detected in faucet samples.

## Abbreviations

The following abbreviations are used in this manuscript:

16S rRNA	16S ribosomal RNA
ASV	Amplicon Sequence Variant
DNA	Deoxyribonucleic Acid
MiSeq	Illumina MiSeq sequencer
PCoA	Principal Coordinates Analysis
PD	Faith’s Phylogenetic Diversity
PERMANOVA	Permutational Multivariate Analysis of Variance
H	Value of test statistic—Kruskal–Wallis H statistic (Kruskal–Wallis test) or pseudo-F (PERMANOVA)
QIIME 2	Quantitative Insights Into Microbial Ecology 2
q2-cutadapt	QIIME 2 plugin for primer/adapter trimming
q2-dada2	QIIME 2 plugin for denoising/dereplication (DADA2)
q2-demux	QIIME 2 plugin for demultiplexing/quality control
q2-sidle	QIIME 2 plugin implementing SMURF for multi-region integration
q2-diveristy	QIIME 2 plugin for calculating metrics and statistics for alpha and beta diversity
R	R statistical computing environment
SMURF	Short MUltiple Reads Framework
V3V4	Hypervariable regions 3–4 of the 16S rRNA gene
V7V9	Hypervariable regions 7–9 of the 16S rRNA gene
